# Automated recognition of major depressive disorder from cardiovascular and respiratory physiological signals

**DOI:** 10.3389/fpsyt.2022.970993

**Published:** 2022-12-09

**Authors:** M. Sami Zitouni, Shu Lih Oh, Jahmunah Vicnesh, Ahsan Khandoker, U. Rajendra Acharya

**Affiliations:** ^1^College of Engineering & IT, University of Dubai, Dubai, United Arab Emirates; ^2^Health Engineering Innovation Center, Khalifa University, Abu Dhabi, United Arab Emirates; ^3^School of Engineering, Ngee Ann Polytechnic, Singapore, Singapore; ^4^Department of Biomedical Engineering, Khalifa University, Abu Dhabi, United Arab Emirates; ^5^Department Bioinformatics and Medical Engineering, Asia University, Taichung City, Taiwan; ^6^International Research Organization for Advanced Science and Technology, Kumamoto University, Kumamoto, Japan; ^7^Department of Biomedical Engineering, School of Science and Technology, SUSS University, Singapore

**Keywords:** major depressive disorder, discrete wavelet transform, nonlinear features, support vector machines, marginal fisher analysis, ECG, pulse, respiratory

## Abstract

Major Depressive Disorder (MDD) is a neurohormonal disorder that causes persistent negative thoughts, mood and feelings, often accompanied with suicidal ideation (SI). Current clinical diagnostic approaches are solely based on psychiatric interview questionnaires. Thus, a computational intelligence tool for the automated detection of MDD with and without suicidal ideation is presented in this study. Since MDD is proven to affect cardiovascular and respiratory systems, the aim of the study is to automatically identify the disorder severity in MDD patients using corresponding multi-modal physiological signals, including electrocardiogram (ECG), finger photoplethysmography (PPG) and respiratory signals (RSP). Data from 88 subjects were used in this study, out of which 25 were MDD patients without SI (MDDSI−), 18 MDD patients with SI (MDDSI+), and 45 normal subjects. Multi-modal physiological signals were acquired from each subject, including ECG, RSP, and PPG signals, and then pre-processed. Discrete wavelet transform (DWT) was applied to the signals, which were decomposed up to six levels, and then eleven nonlinear features were extracted. The features were ranked according to the analysis of variance test and Marginal Fisher Analysis was employed to reduce the feature set, after which the reduced features were ranked again to select the most discriminatory features. Support vector machine with polynomial radial basis function (SVM-RBF) as well as k-nearest neighbor (KNN) classifiers were used to classify the significant features. The performance of the classifiers was evaluated in a 10-fold cross validation scheme. The best performance achieved for the classification of MDDSI+ patients was up to 85.2%, by using selected features from the obtained multi-modal signals with SVM-RBF, while it was up to 96.6% for the detection of MDD patients against healthy subjects. This work is a step toward the utilization of automated tools in diagnostics and monitoring of MDD patients in a personalized and wearable healthcare system.

## 1. Introduction

Suicidal ideations, negative feelings, persistent feelings of sadness and worthlessness or a sense of abyss, are all tell-tale signs of an underlying medical condition known as depression. Depression is a mental disorder defined by the thoughts, feelings and behavior of a person. Depression is multifactorial and is thus triggered by a wide range of factors including genetics, hormonal, environmental, influence of family and socio-cultural aspects (1, 2). The World Health Organization states that depression is a common illness worldwide, where approximately 280 million people of all ages are affected, and it is reported to be leading causes of ill-health and disability (3). The gold standard for depression diagnosis involves a combination of questionnaires and clinical interviews. The interviews range from the composite international diagnostic interview (4), to structured clinical interview for diagnostic and statistical manual of mental disorders. However, this instrument is long-winded and hence undesirable for administration. On the other hand, norm-referenced scales that link score ranges to the severity of symptoms are being used. However, these measures are identified to have poor discriminative ability (5). Hence, a Computational Intelligence Tool (CIT) that is more competent in the accurate and rapid diagnosis of depression is desired.

Various metabolic or neurological disorders such as depression are linked to changes in the functions of the cardiovascular and respiratory systems (6). In our previous study (7), higher breathing frequency and lower amplitude of Respiratory Sinus Arrhythmia (RSA) were found in the MDD patients with SI as compared to the MDD patients without SI, as well as the healthy subjects. Therefore, the resting mental state in MDD patients exerts an influence on RSA oscillations with respect to respiratory movements. Additionally, suicidal ideation in depression was reported to be linked with arterial stiffness as measured by PPG wave parameters that were extracted from fingertip PPG signals (8). The effectiveness of multi-lag Tone–Entropy analysis in identifying MDD patients with Suicidal Ideation was also demonstrated and the alteration in autonomic nervous system modulation of the heart rate related to depression and suicidal ideation was highlighted in another previous study (9).

Thus, in this work, we uniquely propose to combine three types of signals: electrocardiograms (ECGs), pulse or photoplethysmography (PPG), and respiratory signals (RSP) for the detection of depression in a multi-modal classification framework. This study hypothesizes that patients can be classified based on suicidal ideation presence using a multi-modal classification model with combination of ECG, respiration, and PPG signals. This is validated through automated classification of subjects into three classes of MDDSI ± and Healthy using SVM-RBF and nonlinear features in subject independent manner. The comparative study of the proposed method proves the superiority of the multi-modal method in comparison to the commonly used ECG based single-modal methods. The developed technique can be employed with wearable technology as a diagnostic device to help physicians in the diagnosis of MDD with SI and without SI.

## 2. Background

Table 1 presents a summary of literature works using CIT with physiological signals for automated detection and classification of depression. Puthankattil and Joseph (10) employed discrete wavelet transform (DWT) to pre-processed electroencephalogram (EEG) signals and decomposed them to eight levels. Relative wavelet energy features were extracted from the decomposed signals and were used to train and test the artificial neural network, achieving a classification accuracy of 98.11%. Ahmadlou et al. (11) employed the wavelet filter bank (WFB) to decompose the acquired EEG signals, after which the Katz and Higuchi fractal dimensions were computed. The fractal dimension that reflected the greatest difference was fed to a probabilistic neural network (PNN) classifier, yielding to an accuracy of 91.3%. In another study, Ahmadlou et al. (12) similarly decomposed the acquired EEG signals of male and female depressed patients into 5 sub-bands. Spatiotemporal analysis of relative convergence(STARC) was performed to compute the union of loci pair EEGs in the sub-bands and full bands. Large differences in relative convergence of EEG signals were reported in the intra left temporal and front to left temporal lobes between male and female patients. Hosseinifard et al. (13) computed the power of four EEG bands and nonlinear features such as correlation dimension, Higuchi, detrended fluctuation analysis, and large Lyapunov exponent. The resulting feature vector was fed to a KNN classifier, as well as linear discriminant analysis (LDA) and logistic regression classifiers. The highest accuracy of 90% was achieved with the logistic regression classifier combined with the nonlinear features.

**Table 1:** Summary of literature works in automated detection and classification of depression.

**Work**	**Features (number)**	**Methodology**	**Dataset**	**Results (accuracy)**
Khandoker et al. (9)	Tone, entropy features	Mann–Whitney test Classification-regression tree Leave-one-out validation	Healthy: 29 subjects Depression: 16 SI+ & 16 SI- patients	94.83% (SI+)
Puthankattil and Joseph (10)	Nonlinear relative wavelet energy features, (20)	EEG signals Feedforward neural network DWT	Depression: 30 patients	98.11%
Ahmadlou et al. (11)	Katz's fractal dimension and Higuch's fractal dimension nonlinear features, (3)	EEG signals PNN, ANOVA test, WFB Random selection	Healthy: 12 subjects Depression: 12 patients	92.30%
Ahmadlou et al. (12)	Nonlinear spatiotemporal analysis of relative convergence features, (4)	EEG signals WFB	Depression: 22 patients	Differences in relative convergence in intraleft temporal and front to left temporal lobes between genders.
Hosseinifard et al. (13)	Detrended fluctuation analysis, Higuchi fractal, correlation dimension and Lyapunov exponent nonlinear features	Power of EEG bands Logistic regression classifier	Healthy: 45 subjects Depression: 45 patients	90.00%
Faust et al. (14)	Nonlinear parameters	EEG signals frequency bands Wavelet packet decomposition *T*-test PNN	Depression: 30 patients	99.50%
Acharya et al. (15)	Nonlinear features, (15)	EEG signals *T*-test SVM classifier	Healthy: 15 subjects Depression: 15 patients	Development of diagnosis index 98.00%
Sharma et al. (16)	Wavelet sub-band features, (7)	EEG signals Orthogonal WFB *T*-test SVM classifier	Healthy: 15 subjects Depression: 15 patients	99.58%
Bairy et al. (17)	Nonlinear features with skewness, standard deviation, (7)	EEG signals DWT *T*-test SVM-RBF classifier	Healthy: 2,159 samples Depression: 2,400 samples	88.90%
Cai et al. (18)	Linear and nonlinear features, (270)	EEG signals DWT KNN classifier	Healthy: 121 subjects Depression: 92 patients	79.27%
Li et al. (19)	Nonlinear features, (9)	EEG signals Autoregressive model Differential evolution KNN classifier	Healthy: 10 subjects Depression: 10 patients	96.00%
Mantri et al. (20)	Q,S,T features of ECG signal, (3)	ECG signals Principal component analysis		Depression into 4 classes of stress
Kan and Kashihara (21)		ECG signals Spline interpolation Linear onterpolation	ST depression: 25 samples 9 Right before VF 8 During VF 8 Abnormal T waves	cc was higher than T wave abnormality, but lower, before and during VF
Hartmann et al. (22)	Time and frequency domain features	HRV signals ANOVA test	Healthy: 65 subjects Depression: 62 patients	HRV features are related to depression
Byun et al. (23)	Nonlinear and Poincare plot features, (20)	HRV signals Recursive feature elimination Statistical filter SVM classifier	Healthy: 41 subjects Depression: 37 patients	74.40%
Ay et al. (24)	Deep local features from EEG signals	EEG signals CNN-LSTM model	Healthy: 15 subjects Depression: 15 patients	Left hemisphere: 99.12% Right hemisphere: 97.66%
Acharya et al. (7)		EEG signals CNN model	Healthy: 15 subjects Depression: 15 patients	96.00%
Čukić et al. (25)	Higuchi's Fractal Dimension, Sample Entropy, (2)	EEG signals Seven conventional classifiers	Healthy: 20 subjects Depression: 23 patients	90.24–97.56%
Bachmann et al. (26)	Spectral asymmetry index, detrended fluctuation analysis, (2)	EEG signals, Single channel Linear discriminant analysis	Healthy: 17 subjects Depression: 17 patients	91.2%
Avots et al. (27)	Linear, nonlinear EEG features	EEG signals Ensemble classifier	Healthy: 10 subjects Depression: 10 patients	90.24–97.56%
Bachmann et al. (28)	Linear, (3), nonlinear, (3), EEG features	EEG signals Logistic regression classifier	Healthy: 13 subjects Depression: 13 patients	Leave-one-out cross validation, 77–92%

Faust et al. (14) selected suitable frequency bands from EEG signals using wavelet packet decomposition. Nonlinear parameters were subsequently computed from these bands. The significant parameters were selected using *t*-test and then fed to several classifiers. PNN outperformed the other tested classifiers, achieving a classification accuracy of 99.5%. Acharya et al. (15) used brain signals to extract 15 nonlinear parameters and then ranked them through a *t*-test. The optimal features were then fed to SVM, KNN, Naïve Bayes (NB), PNN, and decision tree (DT) classifiers. SVM with polynomial kernel of order 3 yielded the highest accuracy of 98%. A depression diagnosis index was also developed using the discriminatory nonlinear features. An orthogonal WFB with three channels was developed by Sharma et al., to decompose processed EEG signals into seven wavelet sub-bands. Subsequently, L2 norm was estimated for each wavelet sub-band. The resulting feature set was ranked using *t*-test and fed to several classifiers. The SVM classifier performed the best with an accuracy of 99.58% in comparison to other tested techniques.

Bairy et al. (17) employed DWT up to two levels to the acquired EEG signals and then Linear features were extracted. *T*-test was used to select a set of highly significant features. The feature set was fed to an SVM-RBF classifier. SVM-RBF yielded to a classification accuracy of 88.92%. Cai et al. (18) employed DWT to pre-processed EEG signals, after which 270 linear and nonlinear parameters were extracted. The minimal redundancy maximal relevance feature selection technique was subsequently utilized for dimensionality reduction. The reduced feature set was fed to four classifiers, wherein, the highest accuracy of 79.27% was achieved using KNN classifier. Li et al. (19) extracted nine nonlinear features and nine linear features from pre-processed of EEG, using auto regressive model. Differential evolution (DE) was then employed to optimize the features, after which, they were fed to a KNN classifier. DE coupled with KNN yielded an average accuracy of 96%, which was a great improvement in comparison to using only KNN. Mantri et al. (20) computed the Q, S, and T signals from the R peaks of the collected ECG signal. Principal component analysis (PCA) was implemented to extract features from the signals. The extracted values were compared against the expert values for the classification of depression. The proposed method was for classifying depression into four classes of stress, namely hyper acute, acute, hyper chronic and chronic stress.

Cross validation analysis was performed in (21) to compute correlation coefficient (cc) between ST depression and other maladies of varying data points. Through spline interpolation, the cc in the ST depression was found to be higher in T wave abnormality, but lower, in the case of before and during ventricular fibrillation (VF). Additionally, by using linear interpolation, the cc before or during VF was lower than that in ST depression. Hartmann et al. (22) computed time and frequency domain features from pre-processed heart rate variability (HRV) signals using linear and spectral analyses. Nonlinear HRV parameters were estimated using Poincare plots. Analysis of variance (ANOVA) was utilized feature ranking. The proposed method reports a link between HRV parameters and depression, where obvious distinction exists between depressed and healthy individuals. Byun et al. (23) estimated 20 HRV parameters (6 time domain, 7 frequency domain, 5 nonlinear, and 2 Poincare plot features) from the EEG recordings. SVM-recursive feature elimination and statistical filter were used to select the significant features, after which, SVM classifier was used achieving an accuracy of 74.4%. Ay et al. (24) used a developed convolutional neural network (CNN) with raw EEG signals to extract feature maps. The resulting feature maps were fed to the long short-term memory(LSTM) model. Random splitting and 10-fold cross validation techniques were employed to evaluate the CNN-LSTM model, achieving a high accuracy of 99.12 and 97.66% for depression detection in the right and left hemispheres, respectively. Khandoker et al. (9) computed tone and entropy values for multiple logs, from pre-processed HRV signals. The Mann-Whitney test was subsequently employed to compare between healthy and depressed patients. A classification and regression tree was then built for classification. A performance of 94.83% was achieved for the prediction of MDDSI+ patients. Acharya et al. (7) proposed an end-to-end CNN based framework, using raw EEG signals for training and testing. The achieved accuracy was 96% for classifying EEG signals from the right hemisphere. Čukić et al. (25) investigated the effectiveness of applying two nonlinear measures on EEG, including Higuchi's Fractal Dimension and Sample Entropy, for detection of patients diagnosed with depression. Seven classifiers were considered, and the results indicated that good classification was achievable with a small number of principal components, where Sample Entropy had the better performance. Llamocca et al. (29) combined the data from regular reports from standard psychiatric interviews, self-reported daily questionnaires, and data obtained from smart watches to train machine learning models for crisis in bipolar depression prediction. Since bipolar depression have more complex dynamics, it was concluded that a personalized approach is needed. Bachmann et al. (26) investigated using linear, spectral asymmetry index, and nonlinear, detrended fluctuation analysis, for detection of depressed subjects with single EEG channel, where up-to 91.2% classification accuracy was achieved. Avots et al. (27) trained various binary classifiers using linear (relative band power, alpha power variability, spectral asymmetry index) and nonlinear (Higuchi fractal dimension, Lempel–Ziv complexity, detrended fluctuation analysis) EEG features, to study the classification of the long-lasting effects of depression. Bachmann et al. (28) analyzed 30-channel EEG signal using linear methods (spectral asymmetry index, alpha power variability, relative gamma power) and nonlinear methods (Higuchi's fractal dimension, detrended fluctuation analysis, Lempel–Ziv complexity). Logistic regression analysis was used for depressive subjects classification with leave-one-out cross-validation. Classification accuracy of 92% was achieved with mixed combination of three linear and three nonlinear measures.

Brain signals, such as EEG and Functional Magnetic Resonance Imaging (fMRI), are deemed the most effective bio-markers for MDD (30), since they are brain disorders. However, as far as the implementation of these physiological signal-based diagnostic platforms in psychiatry is concerned, wearable technology is the most desirable and feasible option. The physiological relationships of multi-modal signals (ECG, PPG, and RSP), which are easily implementable in wearable settings, with MDD and their suicidal ideations, has been validated (8, 9, 31). Consequently, this study focuses mainly on how machine-learning techniques are able to reliably recognize MDD subjects based on these multi-modal signals.

## 3. Materials and methods

### 3.1. Dataset

In this study, resting ECG, respiration, and finger PPG signals collected from 88 subject for a period of 5–10 min (8), were used. Among these subjects, 45 are healthy and 43 are MDD patients. Out of the MDD patients, 18 are without SI (MDDSI+), while 25 have SI (MDDSI−). In each subject's category (MDDSI+, MDDSI−, healthy), 4, 5, and 19 of them were males and their ages' ranges were 35.0 ± 12.3, 34.92 ± 8.14 and 30.69 ± 9.27 years, respectively. Briefly, diagnoses of the MDD were made by using the Mini-International Neuropsychiatric Interview (MINI) version 5 (32); while C module of the MINI was used to assess the severity of SI. Patients with C module scores more than 9 were considered to be MDDSI+ patients. Table 2 summarizes patient's demographics and psychiatric scores.

**Table 2:** Dataset subjects' demographics and psychiatric scores.

**Variable**	**MDDSI+**	**MDDSI−**	**Healthy**	***P*-value**
Subjects	18	25	45	
Gender male (%)	4 (22%)	5 (20%)	19 (42%)	
Age (yrs)	35.00 ± 12.30	34.92 ± 8.14	30.69 ± 9.27	0.00^*a*^
Height (cm)	161.61 ± 6.73	164.74 ± 17.79	161.76 ± 9.65	0.66
BMI (kg/m^2^)	27.21 ± 6.19	26.58 ± 5.13	24.13 ± 3.39	0.03^*a*^
WC (cm)	87.94 ± 15.37	87.41 ± 16.23	77.13 ± 9.43	0.00^*a*^^,^^*b*^
SBP (mmHg)	112.78 ± 13.20	111.74 ± 13.37	111.48 ± 9.92	0.60
DBP (mmHg)	71.11 ± 8.32	72.61 ± 7.52	72.11 ± 7.27	0.87
BDI	37.17 ± 10.85	33.20 ± 12.11	N/A	0.50
GAD7	16.67 ± 6.51	16.04 ± 8.17	N/A	0.36
PHQ-9	20.78 ± 5.00	19.20 ± 10.29	1.73 ± 1.32	0.50
Suicidal score	18.94 ± 6.39	2.00 ± 3.16	N/A	0.00^2^

BMI, Body mass index; WC, waist circumference; SBP, systolic blood pressure; DBP, diastolic blood pressure; PHQ, patients health questionnaire.

a Significant difference between MDDSI+ and Healthy,

b significant difference between MDDSI+ and MDDSI−.

The study was approved by Al Ain District Ethics Committee, wherein written consent was provided by all participants. Diagnoses were made by a consultant psychiatrist including history of SI, using the MINI (32). The clinical depression severity was assessed using the structured interview guide for the Hamilton Depression Rating Scale (HAM-D) (33). Exclusion criteria included: inadequate reading or verbal fluency in Arabic or English; significant impairment to cognitive abilities; other primary psychiatric diagnosis and active medical diagnoses of ischemic heart disease, diabetes, or inflammatory illness currently or within the preceding 2 years. On the other hand, the inclusion criteria included all those who did not fall under these categories and who were diagnosed as MDDSI± at their first visit to the psychiatric clinic. Additionally, included data were recorded from unmedicated MDD patients only. The healthy group was interviewed by the psychiatrist to check whether they had to go through any psychiatric assessment. The healthy subjects in this study were not required to complete MINI interview and other questionnaire since they declared that they had no previous history of psychiatric disease. MDD patients completed valid and reliable self-report ratings of depression (21-item beck depression inventory BDI), anxiety (general anxiety disorder GAD7), and stress severity (patient health questionnaire PHQ-9), while healthy subjects only completed PHQ-9 rating.

### 3.2. Signals pre-processing

**Figure 1:**
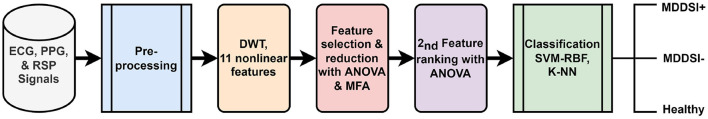
Block diagram of the proposed framework for the recognition of MDD with SI from cardiovascular and respiratory signals.

The three acquired signals for each subject were segmented into 28,800 samples, thus having 3×288000 samples per subject. In order to remove the noise and artifacts from the signals, a 50*Hz* notch filter, as well as a 0.5 − 45*Hz* band-pass filter, were applied. All signals have a sampling rate of 1*kHz*. Figure 1 summarizes the proposed framework.

### 3.3. Feature extraction

It can be deduced from the literature review that DWT and nonlinear feature extraction are commonly used and their suitability is proven for the detection of depression, thus they are adopted in this framework. DWT was applied to decompose each pre-processed signal up to six levels using 6^*th*^ order Daubechies wavelet (db6). In Daubechies (34), the signals pass through low and high pass filters. The filtered signals are subsequently down-sampled to half the maximum frequency, thereby transforming the signals into low pass (approximate) and high pass (detail) coefficients. The same process is repeated to obtain more levels of decomposition. Moreover, Nonlinear features have been widely used to analyze physiological signals, such as ECG (35). Thus, in this framework, eleven nonlinear features that are highly discriminatory, were extracted from each of the 12 DWT coefficients. The nonlinear features used in this work include: 1. approximate entropy (36), 2. signal energy (37), 3. Tsallis entropy (38), 4. Kolmogorov Sinai entropy (39), 5. Rényi entropy (40), 6. Shannon entropy (41), 7. wavelet entropy (42), 8. signal activity (43), 9. Hjorth complexity and mobility (44, 45), 10. Bispectrum (46), and 11. Cumulant (47) features. The features are described follows,

**1. Approximate entropy (Ape):** Ape (36) calculates the amount of regularity and the unpredictability of fluctuations over time-series data even with artifacts. It is commonly used to study ECG signals.

**2. Signal energy (Se):** Se (37) is a commonly estimated measurement in engineering. The energy of a signal is defined by the square of the signal amplitude, integral of a squared signal magnitude or the envelope of a squared signal magnitude.

**3. Tsallis entropy (Te):** Te (38) represents the basic form of the Boltzmann-Gibs theory. It is commonly used for statistical calculations in medicine and physics, hence is explored further is this study.

**4. Kolmogorov-Sinai entropy (Kse):** Kse (39) is used to compute the chaos within a system and it controls the maximum amount of information that can be produced by the system.

**5. Rényi entropy (Re):** Re (40) is closely related to Shannon's entropy. The p^*th*^ entropy, known as the Rényi entropy power, is an extension of Shannon's entropy power.

**6. Shannon entropy (She):** She (41) measures the amount of information needed to recognize random samples within a particular probability distribution.

**7. Wavelet entropy (Wae):** Wae (42) is suitable for analyzing features present in non-stationary signals. Wavelet decomposition combined with entropy is used as a parameter to estimate the chaos level of a signal.

**8. Signal activity (Sa):** Sa (43) is computed based on the number of high frequency components in a system. It describes the variance of time.

**9. Hjorth complexity and mobility (Hcm):** Hcm (44, 45) are useful in the representation of biological signals. These parameters signify statistical properties in signal processing and are prevalently used for feature extraction.

**10. Bispectrum (Bi):** Bi (46) is recognized as a higher order spectra feature that is created through signal decomposition. This feature is used to study nonlinear systems.

**11. Cumulant (Cu):** The Cu (47) of a probability distribution represents the numbers that provide a substitute to the moments of a distribution. This feature is competent in the analysis of imaginary signals.

### 3.4. Feature selection and reduction

In order to select highly significant features, ANOVA test (48) was utilized. Features of *p*-values greater than 0.05 were discarded, while the remaining features were reduced to a smaller dataset using Marginal Fisher Analysis (MFA) (49). The PCA, LDA, and MFA are data reduction techniques, commonly considered in classification problems. PCA, an unsupervised technique, is specially useful in representation and reconstruction. However, it is less effective in discriminating one class from another. In contrast, LDA is a supervised algorithm that emphasizes on the best transformation, by mapping data into a lower dimensional space so that the distribution within the class is minimized, while maximizing the distribution between classes, hence enabling the extraction of most discriminant features (50). Although LDA is considered a better algorithm compared to PCA for solving pattern classification problems, it is still undermined by MFA. MFA is a supervised, diverse learning algorithm that is widely used for the face recognition problems. PCA and LDA work by considering only the global Euclidean structure unlike MFA, which determines the local manifold structure hidden in the high-dimensional data (24). Thus, MFA is believed to be advantageous, due to its competency in providing the characteristics of intraclass spatial arrangement and interclass disconnectedness (11), and is hence adopted in this work. The reduced dataset was then subjected to second iteration of ANOVA test for ranking of the reduced feature set. After the first ANOVA test, there were 309 features, while, the second ANOVA test after feature reduction, led to 9 significant features. Feature selection and reduction methods were performed on data, that was lately used for training the classifier. Table 3 presents the features that were ranked using ANOVA after MFA feature reduction, along their mean and standard deviation (SD) values for each category. As it can be seen in the table, feature ranking was performed based on the estimated statistical components, i.e., their statistical significance. Thus, the feature ranked first has the lowest *p*-value, while the last ranked feature has the highest *p*-value. Hence, the features ranked from 1 to 9 are selected as the best performing features, due to their *p*-values being less than 0.05.

**Table 3:** Range (Mean ± standard deviation) of features ranked using ANOVA after Marginal Fisher Analysis (MFA) feature reduction.

**Rank**	**Healthy**		**MDDSI**−		**MDDSI+**			
	**Mean**	**SD**	**Mean**	**SD**	**Mean**	**SD**	***P*-value**	***F*-value**
1	27.8548	0.0581	27.9438	0.0422	27.9570	0.0309	4.31E-13	40.5522
2	−7.8758	0.0447	−7.9240	0.0383	−7.8981	0.0310	3.81E-05	11.498
3	15.1592	0.0256	15.1211	0.0353	15.1363	0.0454	5.47E-05	11.0402
4	−12.6348	0.0447	−12.6149	0.0506	−12.5835	0.0249	0.0002	9.086
5	−10.3331	0.0399	−10.2927	0.0591	−10.3479	0.0391	0.0002	9.0758
6	12.3819	0.0362	12.3972	0.0530	12.4300	0.0443	0.0006	8.0024
7	−34.1708	0.0287	−34.1610	0.0342	−34.1413	0.0268	0.0029	6.2344
8	0.9622	0.0309	0.9519	0.0550	0.9323	0.0383	0.0342	3.5103
9	−67.1131	0.0374	−67.1397	0.0380	−67.1255	0.0544	0.0405	3.3282
10	−20.7532	0.0581	−20.7823	0.0331	−20.7735	0.0442	0.0522	3.0576
11	−20.3718	0.0619	−20.4002	0.0511	−20.3855	0.0363	0.1180	2.1913
12	68.8739	0.0499	68.8989	0.0572	68.8722	0.0536	0.1294	2.0942
13	31.5075	0.0320	31.4913	0.0436	31.4935	0.0300	0.1319	2.0742
14	34.9067	0.0393	34.9229	0.0377	34.9118	0.0259	0.2127	1.5763
15	−8.1435	0.0339	−8.1371	0.0456	−8.1553	0.039	0.3158	1.1683
16	−16.1624	0.0792	−16.1720	0.0452	−16.1429	0.0487	0.3552	1.0476
17	13.4162	0.0431	13.4202	0.0454	13.4098	0.0456	0.7500	0.2886
18	−12.9198	0.0372	−12.9158	0.0526	−12.9129	0.0970	0.9040	0.1010

### 3.5. MDDSI classification

In this framework, the classification is based on SVM-RBF and KNN classifiers. SVM is often used in binary classification problems due to its ability to map data into a higher dimensional space using a kernel function and generate a linear optimal separating hyper plane between classes (51). The RBF kernel works by mapping samples nonlinearly into a higher dimensional space, making it more competent than linear kernels, in managing cases where the class labels and attributes have a nonlinear relationship (52). On the other hand, the KNN classifier distinguish between features based on the class that is most prevalent among its k nearest neighbors (53). The KNN classifier has been successfully employed for classification tasks involving ECG signals (54–56), achieving promising performances. Moreover, SVM-RBF and KNN classifiers are being widely utilized for the recognition of brain diseases, such as epilepsy (57–59), depression (60), Alzheimer's disease (61), and schizophrenia (62). Subsequently, the SVM-RBF and KNN classifiers were considered in this study. Therefor, the optimal feature set obtained from the feature selection and reduction process was then fed into SVM and KNN classifiers for MDDSI classification.

## 4. Results and discussion

To gauge the MDDSI classification performance, 10-fold cross validation (63) was used, wherein 90% of the data was used for training and 10% for testing, and then performance metrics where estimated, including accuracy (ACC), sensitivity (SEN), specificity (SPC), and positive predictive value (PPV). Table 4 presents the classification results achieved with the SVM-RBF and KNN classifiers in different setups, using selected features from all 3 signals. Table 5 shows the best classification results achieved using both classifiers with single-modal signals, including ECG, PPG, and RSP, along the used feature subset and number. Comparing the results in Table 4, it can be observed that the highest accuracy of 85.22% was achieved using SVM-RBF with 6 features of multi-modal signals. Furthermore, it is noteworthy that SVM-RBF outperformed the KNN classifier in multi-modal analysis, achieving its best performance while only requiring 6 features, against the KNN inferior peak performance of 75.00%, that required 9 features. In contrast, single-modal analysis using ECG, PPG, and RSP, only yielded to peak accuracies of 72.2%, 61.3%, 61.3%, respectively, as reported in Table 5. Additionally, taking into consideration the results obtained from each classifier on every single signal, a higher accuracy was obtained with the SVM-RBF classifier as compared to KNN for ECG, with more features used for training and testing. As for PPG, a higher accuracy was achieved using KNN as compared to SVM-RBF, with 3 more features used. The SVM-RBF classifier resulted in a superior performance using RSP signal, although both were trained and tested with the same number of features.

**Table 4:** MDDSI classification tests results using KNN and SVM-RBF classifiers with features from the 3 physiological signals.

**Classifier**	**Parameters**	**No. of features**	**Feature subset**	**ACC**	**PPV**	**SEN**	**SPC**
KNN	*K* = 10	2	1 to 2	0.5909	0.7879	0.6047	0.8444
	*K* = 10	3	1 to 3	0.5909	0.7813	0.5814	0.8444
	*K* = 5	4	1 to 4	0.6932	0.7561	0.7209	0.7778
	*K* = 5	5	1 to 5	0.7273	0.7949	0.7209	0.8222
	*K* = 5	6	1 to 6	0.7273	0.7949	0.7209	0.8222
	*K* = 5	7	1 to 7	0.7273	0.8333	0.6976	0.8666
	*K* = 5	8	1 to 8	0.7159	0.8333	0.6977	0.8666
	***K* = 5**	**9**	**1 to 9**	**0.7500**	**0.8205**	**0.7442**	**0.8444**
SVM-RBF	σ = 0.1	2	1 to 2	0.5909	0.7179	0.6511	0.7556
	σ = 1.8	3	1 to 3	0.6363	0.7167	1.0000	0.6222
	σ = 0.7	4	1 to 4	0.7954	0.8478	0.9069	0.8444
	σ = 1.5	5	1 to 5	0.7613	0.8431	1.0000	0.8222
	**σ = 1.8**	**6**	**1 to 6**	**0.8522**	**0.9545**	**0.9767**	**0.9555**
	σ = 2.4	7	1 to 7	0.7727	0.8571	0.9767	0.8444
	σ = 2.1	8	1 to 8	0.7386	0.8367	0.9534	0.8222
	σ = 2.9	9	1 to 9	0.8182	0.9318	0.9535	0.9333

*K* is the number of nearest neighbors. σ is the bandwidth of kernel function. Number of ranked features used and their selected subset are According to Table 3. The best results are in bold.

**Table 5:** Top results obtained for MDDSI classification using KNN and SVM-RBF classifiers with single physiological signal.

**Classifier**	**Signal**	**Parameters**	**No. of features**	**Feature subset**	**ACC**	**PPV**	**SEN**	**SPC**
KNN	ECG	*K* = 10	2	1 to 2	0.6932	0.7955	0.8139	0.8000
	PPG	*K* = 5	5	1 to 5	0.6136	0.8000	0.6512	0.8444
	RSP	*K* = 10	3	1 to 3	0.5795	0.6944	0.5814	0.7556
SVM-RBF	ECG	σ = 1.3	3	1 to 3	0.7273	0.8181	0.8372	0.8222
	PPG	σ = 0.5	2	1 to 2	0.5341	0.6604	0.8139	0.6000
	RSP	σ = 1.9	3	1 to 3	0.6136	0.6875	0.7674	0.6667

*K* is the number of nearest neighbors. σ is the bandwidth of kernel function. Number of ranked features used and their selected subset are according to Table 3.

**Figure 2:**
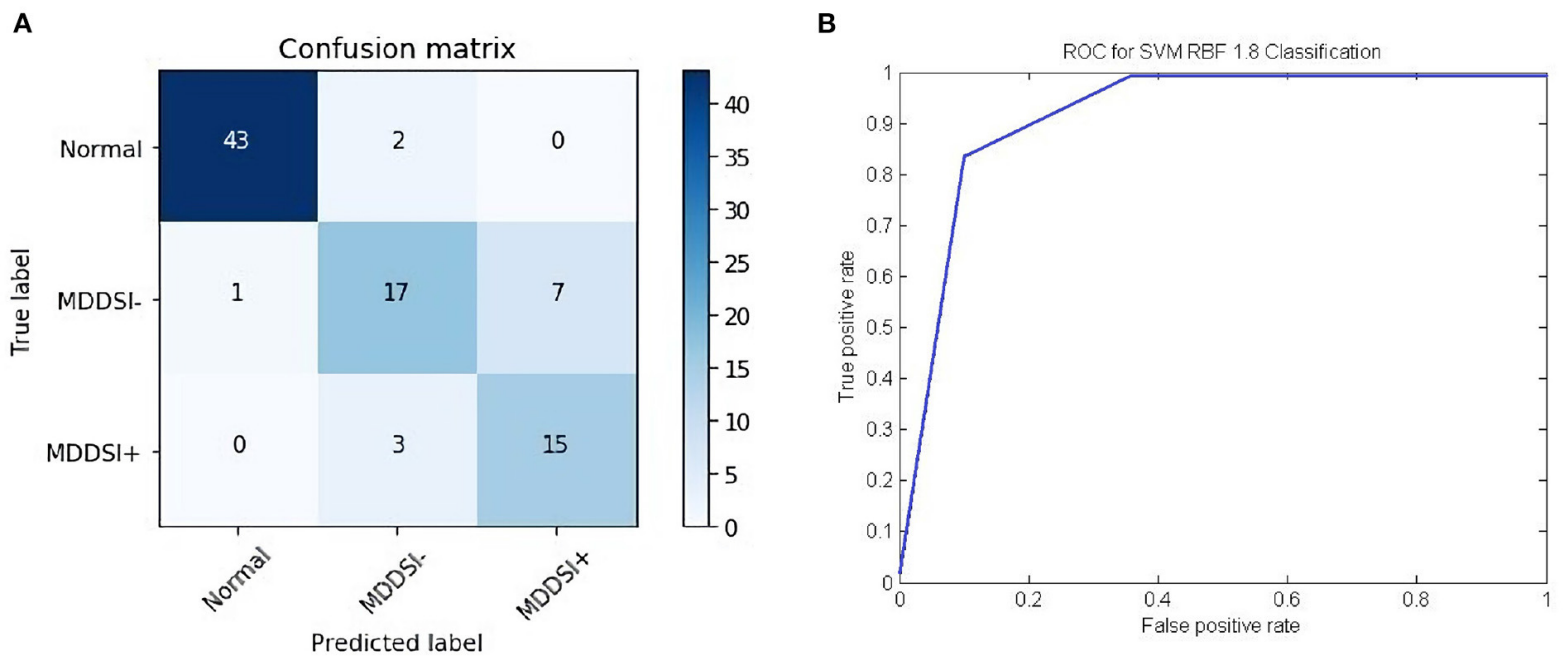
The results of **(A)** confusion matrix, and **(B)** ROC curve, correspond to the best reported results obtained using the SVM-RBF classifier, highlighted in Table 4.

**Figure 3:**
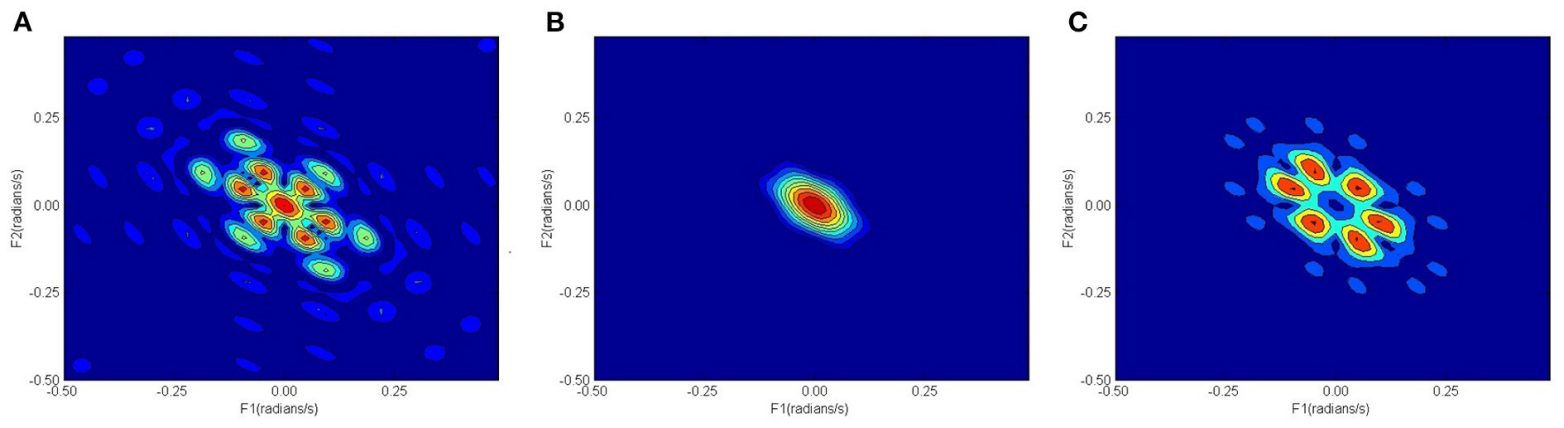
Bispectrum plots, **(A)** normal, **(B)** MDDSI−, **(C)** MDDSI+ ECG signals.

**Figure 4:**
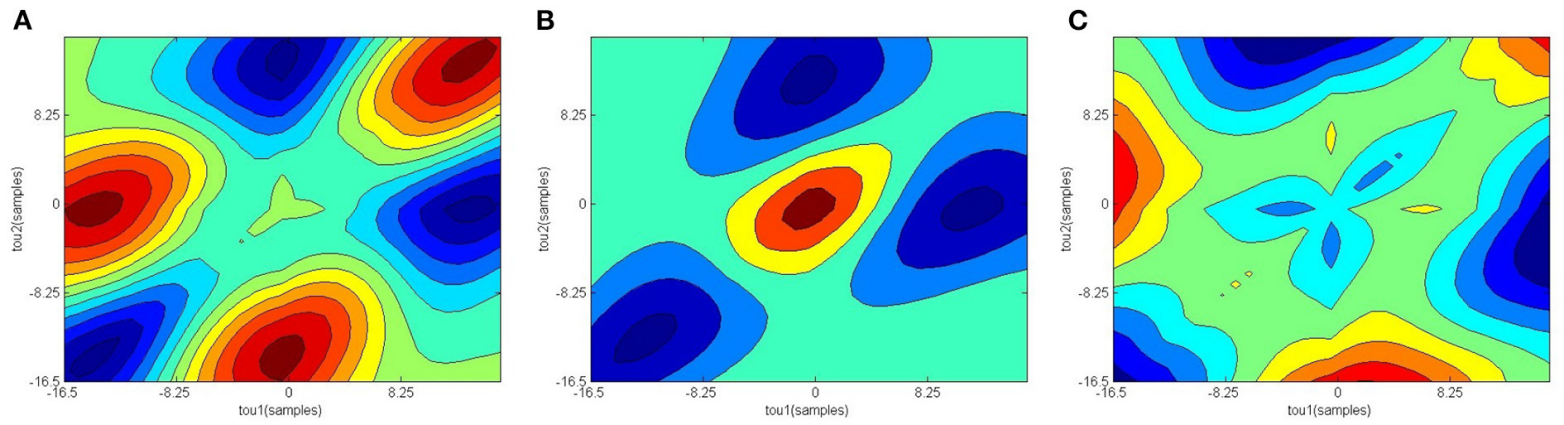
Cumulant plots, **(A)** normal, **(B)** MDDSI−, **(C)** MDDSI+ ECG signals.

Figure 2 shows the confusion matrix of the proposed framework as well as the ROC curve, corresponding to the best reported results using SVM-RBF as in Table 3. It can be estimated from the matrix that there was 4.44% of normal misclassification, 32% as MDDSI−, and 16.7% as MDDSI+. The low misclassification rates verify the robustness of the MDDSI classification framework. The lower classification accuracy achieved for the MDDSI− group in comparison to the other two groups can be explained by the existence of greater overlap between values of MFA features for the MDDSI− groups and the other two groups, as can be observed from the their mean values in Table 1. Overlapping of features depends on the type of features used. Further, from the confusion matrix, it can be estimated that the accuracy for detecting MDD patients against healthy subjects is 85.2% in two classes classification task. Additionally, the ROC curve highlights the robustness of the proposed method. Figure 3 displays the bispectrum plots of (a) normal, (b) MDDSI− and (c) MDDSI+ ECG signals. Moreover, Figure 4 displays the cumulant plots of (a) normal, (b) MDDSI−, and (c) MDDSI+ ECG signals as well. As can be seen in both Figures 3, 4, it is observable that the bispectrum and cumulant patterns are distinctive and unique for each MDDSI class. This attests that the nonlinear features used and the patterns obtained are highly discriminatory for the presented classification task. Additionally, this proves that the selected features are distinctive for recognizing the MDD cases from the three collected physiological signals.

To compare the presented study and results against the state-of-the-art, the summary in Table 1 is observed. First, it can be noticed that works, including (10), (11), (13), (14), (15), (16), (24), and (7) achieved high accuracies of above 90%. However, these studies mainly use brain EEG signals, and, except for (13), they involved low number of subjects compared to the presented study. Additionally, these studies only perform 2-class classification, for which 96.6% was achieved in this study without EEG, making study more promising. Both Mantri et al. (20) and Kan and Kashihara (21) explored the use of ECG signals, but did not report any classification performance. Similarly, Hartmann et al. (22) explored the use of HRV signals, but only reported a link between HRV features and depression without classification. Byun et al. (23) achieved a low classification performance using HRV parameters. Further, Khandoker et al. (9) examined HRV signals and was able to achieve higher accuracy for predicting MDD with SD. However, this study included a smaller number of subjects, and the classification method involved the use of personal data, such as age, body mass index and waist circumference, which can be highly discriminative when low number of subjects are used.

Hence, as per the literature review and comparison, this is the first study to investigate the classification of MDDSI (3 classes) using a multi-modal combining ECG, PPG, and RSP signals. Since the cardiovascular system is interconnected with heart, blood vessels, and respiratory system, combining these specific signals allowed the proposed framework to perform effectively, while being physiologically relevant. This validates the study's hypothesis that patients can be classified based on suicidal ideation presence using a multi-modal classification model with the aforementioned signals. Additionally, MFA based features were discriminative in MDDSI classification task due to potentially containing information of interactions between cardiovascular and respiratory systems through these signals. Further, mental state in MDD patients had an influence on respiratory sinus arrhythmia oscillations, where the amplitudes decrease, inducing incoherent phase lag with respect to respiratory movements, as was shown in a previous study (31). Further, in this work, the nonlinear features were extracted for each subject's signals using their entire recordings. Thus, the number of samples was insufficient to train deep learning models, such as in (64, 65), since over-fitting will be significant. On the other hand, the obtained results are very promising since the validation was conducted in a subject independent manner, where data of subjects used for training are excluded from testing. This demonstrates a considerable advantage against works using deep learning in subject dependent manner.

## 5. Conclusion

Depression is a mental disorder that makes a human overwhelmed with sadness or negative feelings for long periods of time, which ends up triggering suicidal thoughts. Conventional diagnostic approaches for depression either include interviews that are too long-winded, or use norm-referenced scales that poorly discern depression from normal. Currently, tell-tale signs of suicidal risk are largely subjective and could be overlooked by individuals, and in some cases even experts, which potentially leads to undesirable outcomes. A CIT can be crucial and highly assistive, thus an automated MDDSI recognition framework was presented. Neurological conditions such as depression are linked to changes in the functions of the cardiovascular and respiratory systems. Hence, ECG, PPG and RSP signals were acquired and processed from each subject. DWT was then employed to obtain DWT coefficients, from which eleven nonlinear features were extracted. ANOVA test was subsequently conducted to rank the features. The ranked features were reduced using MFA. The set of reduced features were then ranked again using ANOVA, to obtain the most significant features. SVM-RBF and KNN classifiers were used for the recognition of the MDDSI class. Ten-fold cross validation was performed to quantify the performance of the classification model. An accuracy upto 85.2% was achieved for 3 classes MDDSI classification (96.6 for 2 classes MDD classification) using the SVM-RBF classifier, with the three acquired signals as inputs. This was proven to be far more superior in comparison to a single-modal based on any individual signal. Thus, this shows the capability of such system to be used as a diagnostic tool to aid clinicians in the detection of depression with SI.

Future studies should involve a much larger cohort of MDD patients with various degrees of suicidal risk evaluations. Further, follow up studies on the outcomes from the same cohort of MDD patients following treatments should be used for validation of the proposed method, so that the development of a system for monitoring the MDD patients on regular basis is facilitated. Therefore, with a larger dataset, an end-to-end deep learning based framework with continuous fine-tuning of hyper-parameters, following the development of the MDDSI patients status, can be investigated. Moreover, Bayesian optimization (64) can be employed to tune the hyper-parameters of the machine learning model to obtain optimal parameter settings and improve its performance. Additionally, future work will investigate the use of deep leaning on shorter data segments not only to identify MDD patients, but also classify and assess the severity of depression and suicidal ideation based on both the acquired psychiatric and self-report ratings of depression, for continuous monitoring and assistive diagnostics. Although the automated recognition of MDD is not at a stage where it can be considered for clinical implementation, the obtained results are promising, and the evolution of the proposed method through the employment of the future plans is very feasible. The clinical interpretation of this work can be achieved through the utilization of such technique as an assistive diagnostic tool to help psychiatrists and enhance the accuracy of their diagnosis, while allowing them to develop personalized treatments considering the continuous monitoring offered through the use of automated recognition with wearable technology, in patients daily life.

## Data availability statement

The raw data supporting the conclusions of this article will be made available by the authors, without undue reservation.

## Ethics statement

The studies involving human participants were reviewed and approved by Al Ain District Ethics Committee. Participants provided written informed consent to participate in the study.

## Author contributions

SL developed the diagnostic system and provided the diagrams. MZ and JV drafted the paper. AK provided the data for study and edited the paper. UA conceptualized the methodology and edited the paper. All authors have read and approved the manuscript.

## References

[1] Bembnowska M Josko-Ochojska J. What causes depression in adults? Polish J Public Health. (2015) 125:116–20. 10.1515/pjph-2015-0037

[2] Kipli K Kouzani AZ Williams LJ. Towards automated detection of depression from brain structural magnetic resonance images. Neuroradiology. (2013) 55:567–84. 10.1007/s00234-013-1139-8 23338839

[3] World Health Organization. Depression. (2021).

[4] Haugen W Haavet OR Sirpal MK Christensen KS. Identifying depression among adolescents using three key questions: a validation study in primary care. Brit J Gen Pract. (2016) 66:e65–70. 10.3399/bjgp16X683461 26823267 PMC4723216

[5] Dunstan DA Scott N Todd AK. Screening for anxiety and depression: reassessing the utility of the Zung scales. BMC Psychiatry. (2017) 17:329. 10.1186/s12888-017-1489-6 28886698 PMC5591521

[6] Kapidzic A Platisa MM Bojic T Kalauzi A. Nonlinear properties of cardiac rhythm and respiratory signal under paced breathing in young and middle-aged healthy subjects. Med Eng Phys. (2014) 36:1577–84. 10.1016/j.medengphy.2014.08.007 25199589

[7] Acharya UR Oh SL Hagiwara Y Tan JH Adeli H Subha DP. Automated EEG-based screening of depression using deep convolutional neural network. Comput Methods Prog Biomed. (2018) 161:103–13. 10.1016/j.cmpb.2018.04.012 29852953

[8] Khandoker AH Luthra V Abouallaban Y Saha S Ahmed KIU Mostafa R. Suicidal ideation is associated with altered variability of fingertip photo-plethysmogram signal in depressed patients. Front Physiol. (2017) 8:501. 10.3389/fphys.2017.00501 28769817 PMC5516215

[9] Khandoker AH Luthra V Abouallaban Y Saha S Ahmed KI Mostafa R. Predicting depressed patients with suicidal ideation from ECG recordings. Med Biol Eng Comput. (2017) 55:793–805. 10.1007/s11517-016-1557-y 27538398

[10] Puthankattil SD Joseph PK. Classification of EEG signals in normal and depression conditions by ANN using RWE and signal entropy. J Mech Med Biol. (2012) 12:1240019. 10.1142/S0219519412400192

[11] Ahmadlou M Adeli H Adeli A. Fractality analysis of frontal brain in major depressive disorder. Int J Psychophysiol. (2012) 85:206–11. 10.1016/j.ijpsycho.2012.05.001 22580188

[12] Ahmadlou M Adeli H Adeli A. Spatiotemporal analysis of relative convergence of EEGs reveals differences between brain dynamics of depressive women and men. Clin EEG Neurosci. (2013) 44:175–81. 10.1177/1550059413480504 23545250

[13] Hosseinifard B Moradi MH Rostami R. Classifying depression patients and normal subjects using machine learning techniques and nonlinear features from EEG signal. Comput Methods Prog Biomed. (2013) 109:339–45. 10.1016/j.cmpb.2012.10.008 23122719

[14] Faust O Ang PCA Puthankattil SD Joseph PK. Depression diagnosis support system based on EEG signal entropies. J Mech Med Biol. (2014) 14:1450035. 10.1142/S0219519414500353

[15] Acharya UR Sudarshan VK Adeli H Santhosh J Koh JEW Puthankatti SD. A novel depression diagnosis index using nonlinear features in EEG signals. Eur Neurol. (2015) 74:79–83. 10.1159/000438457 26303033

[16] Sharma M Achuth PV Deb D Puthankattil SD Acharya UR. An automated diagnosis of depression using three-channel bandwidth-duration localized wavelet filter bank with EEG signals. Cogn Syst Res. (2018) 52:508–20. 10.1016/j.cogsys.2018.07.010

[17] Bairy GM Niranjan U Puthankattil SD. Automated classification of depression EEG signals using wavelet entropies and energies. J Mech Med Biol. (2016) 16:1650035. 10.1142/S0219519416500354

[18] Cai H Han J Chen Y Sha X Wang Z Hu B. A pervasive approach to EEG-based depression detection. Complexity. (2018) 2018:1–13. 10.1155/2018/5238028

[19] Li Y Hu B Zheng X Li X. EEG-based mild depressive detection using differential evolution. IEEE Access. (2019) 7:7814–22. 10.1109/ACCESS.2018.2883480

[20] Mantri S Agrawal P Patil D Wadhai V. Depression analysis using ECG signal. Int J Comput Technol. (2013) 11:2746–51. 10.24297/ijct.v11i7.3470

[21] Kan Y Kashihara K. Automatic detection of ST depression on ECG. In: 2015 IEEE 4th Global Conference on Consumer Electronics (GCCE). Osaka: IEEE (2015). p. 655–7. 10.1109/GCCE.2015.7398704

[22] Hartmann R Schmidt FM Sander C Hegerl U. Heart rate variability as indicator of clinical state in depression. Front Psychiatry. (2019) 9:735. 10.3389/fpsyt.2018.00735 30705641 PMC6344433

[23] Byun S Kim AY Jang EH Kim S Choi KW Yu HY. Detection of major depressive disorder from linear and nonlinear heart rate variability features during mental task protocol. Comput Biol Med. (2019) 112:103381. 10.1016/j.compbiomed.2019.103381 31404718

[24] Ay B Yildirim O Talo M Baloglu UB Aydin G Puthankattil SD. Automated depression detection using deep representation and sequence learning with EEG signals. J Med Syst. (2019) 43:1–12. 10.1007/s10916-019-1345-y 31139932

[25] Čukić M Stokić M Simić S Pokrajac D. The successful discrimination of depression from EEG could be attributed to proper feature extraction and not to a particular classification method. Cogn Neurodyn. (2020) 14:443–55. 10.1007/s11571-020-09581-x 32655709 PMC7334335

[26] Bachmann M Lass J Hinrikus H. Single channel EEG analysis for detection of depression. Biomed Signal Process Control. (2017) 31:391–7. 10.1016/j.bspc.2016.09.010

[27] Avots E Jermakovs K Bachmann M Päeske L Ozcinar C Anbarjafari G. Ensemble approach for detection of depression using EEG features. Entropy. (2022) 24:211. 10.3390/e24020211 35205506 PMC8871180

[28] Bachmann M Päeske L Kalev K Aarma K Lehtmets A Ööpik P. Methods for classifying depression in single channel EEG using linear and nonlinear signal analysis. Comput Methods Prog Biomed. (2018) 155:11–17. 10.1016/j.cmpb.2017.11.023 29512491

[29] Llamocca P López V Čukić M. The proposition for bipolar depression forecasting based on wearable data collection. Front Physiol. (2022) 12:777137. 10.3389/fphys.2021.777137 35145422 PMC8821957

[30] Ke H Cai C Wang F Hu F Tang J Shi Y. Interpretation of frequency channel-based CNN on depression identification. Front Comput Neurosci. (2021) 15:773147. 10.3389/fncom.2021.773147 35027888 PMC8750060

[31] Khandoker A Luthra V Abouallaban Y Widatalla N F Jelinek H Niizeki K. Incoherent synchronization between resting state respiratory sinus arrhythmia and respiratory movement in depressed patients with suicidal ideation. In: 2018 Computing in Cardiology Conference (CinC). Maastricht (2018). 10.22489/CinC.2018.173

[32] Sheehan DV Lecrubier Y Sheehan KH Amorim P Janavs J Weiller E. The Mini-International Neuropsychiatric Interview (M.I.N.I.): the development and validation of a structured diagnostic psychiatric interview for DSM-IV and ICD-10. J Clin Psychiatry. (1998) 59:22–57. 10.1037/t18597-000 9881538

[33] Williams JBW. A structured interview guide for the Hamilton depression rating scale. Arch Gen Psychiatry. (1988) 45:742. 10.1001/archpsyc.1988.01800320058007 3395203

[34] Daubechies I. Ten Lectures on Wavelets. SIAM (1992). 10.1137/1.9781611970104

[35] Jahmunah V Oh SL Wei JKE Ciaccio EJ Chua K San TR. Computer-aided diagnosis of congestive heart failure using ECG signals–A review. Phys Med. (2019) 62:95–104. 10.1016/j.ejmp.2019.05.004 31153403

[36] Pincus SM. Approximate entropy as a measure of system complexity. Proc Natl Acad Sci USA. (1991) 88:2297–301. 10.1073/pnas.88.6.2297 11607165 PMC51218

[37] Oppenheim AV Willsky AS Nawab SH. Signals & Systems. 2nd ed. Delhi: Pearson Education Asia (2014).

[38] Anastasiadis A. Special issue: Tsallis entropy. Entropy. (2012) 14:174–6. 10.3390/e1402017429911318

[39] Pham TD. The Kolmogoro–Sinai entropy in the setting of fuzzy sets for image texture analysis and classification. Pattern Recogn. (2016) 53:229–37. 10.1016/j.patcog.2015.12.012

[40] Savare G Toscani G. The concavity of Renyi entropy power. IEEE Trans Inform Theory. (2014) 60:2687–93. 10.1109/TIT.2014.230934134945899

[41] Vajda S. The Mathematical Theory of Communication. By Claude E. Shannon and Warren Weaver. Pp. 117 $2.50. 1949 (University of Illinois Press, Urbana). Math Gazette. (1950). 34:312–3. 10.2307/3611062

[42] Rosso OA Blanco S Yordanova J Kolev V Figliola A Schurmann M. Wavelet entropy: a new tool for analysis of short duration brain electrical signals. J Neurosci Methods. (2001) 105:65–75. 10.1016/S0165-0270(00)00356-3 11166367

[43] Sweller J. Cognitive load during problem solving: effects on learning. Cogn Sci. (1988) 12:257–85. 10.1207/s15516709cog1202_4

[44] Sweller J van Merrienboer JJG Paas FGWC. Cognitive architecture and instructional design. Educ Psychol Rev. (1998) 10:251–96. 10.1023/A:1022193728205

[45] Brunken R Plass JL Leutner D. Direct measurement of cognitive load in multimedia learning. Educ Psychol. (2003) 38:53–61. 10.1207/S15326985EP3801_7 12053529

[46] Collis W White P Hammond J. Higher-order spectra: the bispectrum and trispectrum. Mech Syst Signal Process. (1998) 12:375–94. 10.1006/mssp.1997.01459203002

[47] Brillinger DR. An introduction to polyspectra. Ann Math Stat. (1965) 36:1351–74. 10.1214/aoms/1177699896

[48] Gelman A. Analysis of variance–why it is more important than ever. Ann Stat. (2005) 33:1–53. 10.1214/009053604000001048

[49] Yan S Xu D Zhang B Zhang HJ Yang Q Lin S. Graph embedding and extensions: a general framework for dimensionality reduction. IEEE Trans Pattern Anal Mach Intell. (2007) 29:40–51. 10.1109/TPAMI.2007.250598 17108382

[50] Wang Z Sun X Sun L Huang Y. Semisupervised Kernel marginal fisher analysis for face recognition. Sci World J. (2013) 2013:1–13. 10.1155/2013/981840 24163638 PMC3791838

[51] Khandoker AH Palaniswami M Karmakar CK. Support vector machines for automated recognition of obstructive sleep apnea syndrome from ECG recordings. IEEE Trans Inform Technol Biomed. (2009) 13:37–48. 10.1109/TITB.2008.2004495 19129022

[52] Apostolidis-Afentoulis V Konstantina-Ina L,. SVM Classification with Linear RBF Kernels. (2015). Available online at: http://www.academia.edu/13811676/SVM

[53] Zhang Z. Introduction to machine learning: k-nearest neighbors. Ann Transl Med. (2016) 4:218–218. 10.21037/atm.2016.03.37 27386492 PMC4916348

[54] Acharya UR Fujita H Adam M Lih OS Sudarshan VK Hong TJ. Automated characterization and classification of coronary artery disease and myocardial infarction by decomposition of ECG signals: a comparative study. Inform Sci. (2017) 377:17–29. 10.1016/j.ins.2016.10.013

[55] Sharma M Tan RS Acharya UR. Automated heartbeat classification and detection of arrhythmia using optimal orthogonal wavelet filters. Inform Med Unlocked. (2019) 16:100221. 10.1016/j.imu.2019.100221

[56] Sridhar C Acharya UR Fujita H Bairy GM. Automated diagnosis of coronary Artery Disease using nonlinear features extracted from ECG signals. In: 2016 IEEE International Conference on Systems, Man, and Cybernetics (SMC). Budapest: IEEE (2016). p. 545–9. 10.1109/SMC.2016.7844296

[57] Sujitha V Sivagami P Vijaya MS. Support vector machine based epilepsy prediction using textural features of MRI. Proc Comput Sci. (2010) 2:283–90. 10.1016/j.procs.2010.11.036

[58] Acharya UR Sree SV Ang PCA Yanti R Suri JS. Application of nonlinear and wavelet based features for the automated identification of epileptic EEG signals. Int J Neural Syst. (2012) 22:1250002. 10.1142/S0129065712500025 23627588

[59] Acharya UR Sree SV Chattopadhyay S Yu W Ang PCA. Application of recurrence quantification analysis for the automated identification of epileptic EEG signals. Int J Neural Syst. (2011) 21:199–211. 10.1142/S0129065711002808 21656923

[60] Acharya UR Sudarshan VK Adeli H Santhosh J Koh JEW Adeli A. Computer-aided diagnosis of depression using EEG signals. Eur Neurol. (2015) 73:329–36. 10.1159/000381950 25997732

[61] Acharya UR Fernandes SL WeiKoh JE Ciaccio EJ Fabell MKM Tanik UJ. Automated detection of Alzheimer's disease using brain MRI images–A study with various feature extraction techniques. J Med Syst. (2019) 43:302. 10.1007/s10916-019-1428-9 31396722

[62] Jahmunah V Lih Oh S Rajinikanth V Ciaccio EJ Hao Cheong K Arunkumar N. Automated detection of schizophrenia using nonlinear signal processing methods. Artif Intell Med. (2019) 100:101698. 10.1016/j.artmed.2019.07.006 31607349

[63] Duda RO Hart PE Stork DG. Pattern Classification, New York: John Wiley & Sons, 2001, pp. xx + 654, ISBN: 0-471-05669-3. J Class. (2007) 24:305–7. 10.1007/s00357-007-0015-9

[64] Ke H Chen D Shi B Zhang J Liu X Zhang X. Improving brain E-health services via high-performance EEG classification with grouping Bayesian optimization. IEEE Trans Serv Comput. (2019) 13:696–708. 10.1109/TSC.2019.2962673

[65] Ke H Chen D Shah T Liu X Zhang X Zhang L. Cloud-aided online EEG classification system for brain healthcare: a case study of depression evaluation with a lightweight CNN. Softw Pract Exp. (2020) 50:596–610. 10.1002/spe.2668

